# Design of High-Performance Molecular Imprinted Magnetic Nanoparticles-Loaded Hydrogels for Adsorption and Photodegradation of Antibiotics from Wastewater

**DOI:** 10.3390/polym16152096

**Published:** 2024-07-23

**Authors:** Giusy Curcuruto, Andrea A. Scamporrino, Roberta Puglisi, Giuseppe Nicotra, Gianfranco Sfuncia, Giuliana Impellizzeri, Sandro Dattilo, Anne Kahru, Mariliis Sihtmäe, Villem Aruoja, Irina Blinova, Sabrina Carola Carroccio

**Affiliations:** 1 Institute for Polymers, Composites, and Biomaterials CNR-IPCB, Via Paolo Gaifami 18, 95126 Catania, Italy; giusy.curcuruto@cnr.it (G.C.); roberta.puglisi@unict.it (R.P.); sandro.dattilo@cnr.it (S.D.); sabrinacarola.carroccio@cnr.it (S.C.C.); 2 Department of Chemical Sciences, University of Catania, Viale Andrea Doria 6, 95125 Catania, Italy; gianfranco.sfuncia@cnr.it; 3 Institute for Microelectronics and Microstructures CNR-IMM, Zona Industriale Strada VIII, 5, 95121 Catania, Italy; giuseppe.nicotra@cnr.it; 4 Institute for Microelectronics and Microstructures CNR-IMM, Via Santa Sofia 64, 95123 Catania, Italy; giuliana.impellizzeri@ct.infn.it; 5 Laboratory of Environmental Toxicology, National Institute of Chemical Physics and Biophysics, Akadeemia tee 23, 12618 Tallinn, Estonia; mariliis.sihtmae@kbfi.ee (M.S.); villem.aruoja@kbfi.ee (V.A.); irina.blinova@kbfi.ee (I.B.)

**Keywords:** hydrogels, degradation, photocatalysis, HEMA, characterization, ecotoxicology

## Abstract

A hydrogel formulation of 2-hydroxy ethyl methacrylate (HEMA) containing covalently linked magnetite nanoparticles was developed to actively facilitate the selective removal and photocatalytic degradation of antibiotics. To this purpose, the hybrid materials were molecularly imprinted with Lomefloxacin (Lome) or Ciprofloxacin (Cipro), achieving a selectivity of 60% and 45%, respectively, starting from a solution of XX concentration. After the adsorption, the embedded magnetite was used with the double function of (i) magnetically removing the material from water and (ii) triggering photo-Fenton (PF) reactions assisted by UVA light and H_2_O_2_ to oxidize the captured antibiotic. The success of the material design was confirmed by a comprehensive characterization of the system from chemical–physical and morphological perspectives. Adsorption and degradation tests demonstrated the material’s ability to efficiently degrade Lome until its complete disappearance from the electrospray ionization (ESI) mass spectra. Regeneration tests showed the possibility of reusing the material for up to three cycles. Ecotoxicological tests using algae *Rapidocelis subcapitata*, crustaceans *Daphnia magna*, and bacteria *Vibrio fischeri* were performed to evaluate the ecosafety of our synthesized materials.

## 1. Introduction

Water is the primary source for flora, fauna, and human life. Considering this undeniable fact, it should be clear that unintentionally or purposely introduced synthetic molecules, which are not part of the natural water cycle, could severely impact all life forms. Despite their low concentrations (µg/L) in water, pollution from antibiotics is increasingly under the spotlight of the United Nations (UN) and scientific communities. It is estimated that antimicrobial drug-resistant diseases could cause 10 million deaths each year by 2050 [1].

Antibiotics, which were overused in the last 20 years in medical and veterinary applications without specific disposal regulations, represent some of the most insidious pharmaceuticals. They are not completely metabolized, and 30–90% of the drugs are excreted into rivers and oceans. Traditional water treatment systems only partially degrade antibiotics or may even leave the drug molecules unchanged [2,3,4].

Waterbodies are, consequently, reservoirs of antibiotics, inducing antibiotic resistance, and thwarting the significant advantages of their invention in the future. In the European Commission’s (EC) checklist of substances to be monitored in the field of water policy [5], there are commonly used antibiotics (such as Ciprofloxacin, Lomefloxacin, and Azithromycin) to treat bacteria-induced diseases (urinary and/or respiratory tract infections, for example). Therefore, new removal strategies to prevent the spread of antibiotics, especially in water bodies, need to be developed. Selectivity towards the target contaminant is a crucial aspect of the design of innovative technologies. However, antibiotics are present in water bodies at extremely low concentrations (typically in micro or nanograms per liter) and coexist with other contaminants. This significantly limits the effectiveness of removal approaches. The adsorption process can readily reach material saturation without thoroughly removing the specific contaminants. Similar constraints are evident in photocatalytic processes. In addition, purification via nanoparticles (ZnO, TiO_2_, MoS_2_, etc.) has a severe technological limit: photocatalyst nanoparticles must be removed after water treatment to avoid threats to aquatic and human health. The molecular imprinted polymer (MIP) technology was applied to enhance the selectivity towards pharmaceuticals. Several papers were published in the last decade that successfully addressed this technology to deal with the pollution from emerging contaminants [6,7,8,9,10,11,12].

However, the adsorbent MIP-powders, which boost removal efficiency, impose time-consuming and cost-effective filtration post-treatment steps. Fe_3_O_4_-MIP nanoparticles were designed to remove the adsorbent magnetically to overcome the abovementioned issues [13,14].

In this case, antibiotics remain entrapped into the materials, generating another concern: the final disposal of stored contaminated materials.

The proof of concept introduced in this work pushes up the limit of the adsorption method by exploiting the photo-Fenton [15,16,17] reaction applied to the imprinted material after contaminant adsorption to degrade the contaminant (i.e., the antibiotic). In this view, magnetite has a double function in the drug removal process: (i) to allow the magnetic removal of drug-saturated MIP from the contaminated water, and (ii) to mineralize the respective contaminant by photo-Fenton reaction, thus restoring the MIP for further use. Specifically, Lome [18] and Cipro [19,20,21] were used as model templating antibiotics. Both antibiotics mentioned above belong to the fluoroquinolones group, the third most significant group of antibiotics sold worldwide in 2009 [22]. As polymeric counterparts, we focused our attention on HEMA-based hydrogel since we already tested its durability under UV light irradiation for different regeneration cycles [23]. The use of MIP linked with magnetite makes the studied system innovative, selective, and efficient. Indeed, the selectivity induced by the MIP allows the effective treatment of large volumes of water, minimizing the saturation of the active sites by non-target pollutants, and the magnetite acts as a trigger for the photo-Fenton and allows rapid and complete removal from the water adsorbent particles using a magnet.

## 2. Materials and Methods

Magnetite (Fe_3_O_4_) nanopowders, 50–100 nm particle primary size (SEM), hydroxyethyl-methacrylate (HEMA), N-N′-methylenebisacrylamide (MBA), ammonium persulfate (APS), tetramethylethylenediamine (TEMED), methylene blue (MB), methyl orange (MO), (trimethoxysilyl)propyl methacrylate, and 2,4-dichlorophenoxyacetic acid (2,4-D), were purchased from Sigma Aldrich (Merk Life Science S.r.l. Milano, Italy)and ZnSO_4_x7H_2_O from Alfa Aesar (Lancashire, UK) and used without any further purification. Lome and Cipro hydrochloride were provided by Medivis (Catania, Italy) and used as received.

The thermal behavior of each sample was investigated through a thermogravimetric analyzer TGA Q500 (TA Instruments, New Castle, DE, USA) under nitrogen flow (with a rate of 60 mL/min) at a heating ramp of 10 °C/min from 50 to 800 °C, analyzing 5 ± 0.1 mg of sample.

The UV–VIS spectra were acquired by a V-750 Spectrometer (Jasco Inc., Easton, MD, USA). Data were processed with the software provided by the manufacturer. 

The morphological investigation was carried out through a scanning electron microscopy (SEM) Phenomenex microscope. Microporous samples (1–2 mm) were dried in a vacuum oven and then given a sputtering with gold (<10 nm) to confer conductivity. The data were acquired and processed using Phenom Porometric 1.1.2.0 (Phenom-World BV, Eindhoven, The Netherlands). Transmission electron microscopy (TEM) analyses were performed using a Jeol ARM-200F (Jeol Ltd Akishima, Tokio, Japan) microscope operating in conventional parallel beam mode (C/TEM) at an acceleration voltage of 200 kV, equipped with a cold field emission gun, a CEOS CESCOR spherical aberration corrector, and an EDS Centurio with 100 mm^2^ SDD detector and energy resolution of 127 eV. A 4k × 4k direct detection Gatan K2 Summit camera acquired TEM images in bright field (BF) mode. The powders obtained by gently mashing the hydrogels were first embedded in resin using the Poly/Bed^®^ 812 (Luft formulations) embedding kit/DMP-30 for sample characterization. The obtained resin blocks were then trimmed using a Leica EM TXP to create a trapezoidal flat block face, which was cut using a Leica EM UC7 ultramicrotome equipped with a Diatome ultra 35° diamond knife to produce 70 nm thin sections, which were deposited on copper TEM grids.

Fourier transform infrared analyses (FTIR) of the whole set of samples were performed in the 4000–400 cm^−1^ range using FTIR System 2000 (Perkin-Elmer, Waltham, MA, USA) and KBr as media.

Electrospray ionization (ESI) mass analyses (MS) were performed by a Thermo Scientific Exactive Plus Orbitrap MS (Thermo Fischer Scientific, San Jose, CA, USA), using a heated electrospray ionization (HESI II) interface. Mass spectra were recorded operating in positive ion mode in the range 100–1000 *m*/*z* at a resolving power of 25,000 *m*/*z* (full-width-at-half-maximum, at 200 *m*/*z*, (FWHM), resulting in a scan rate of >1.5 scans/s when using automatic gain control target of 1.0 × 10^6^ and a C-trap inject time of 100 ms under the following conditions: capillary temperature 300 °C, nebulizer gas (nitrogen) with a flow source voltage 3.5 kV; capillary voltage 82.5 V; tube lens voltage 100 V for positive analysis and capillary temperature 300 °C, nebulizer gas (nitrogen) with a flow rate of 20 arbitrary units; and an auxiliary gas flow rate of 5 arbitrary units. The Lome concentration was determined via direct infusion by using an ultra-high-performance liquid chromatography (UHPLC) system coupled to the Orbitrap MS. Samples (1 µL after appropriate dilution) directly introduced into the mass spectrometer, were subjected to an isocratic elution at a flow rate of 0.1 mL/min using a mobile phase of H_2_O 100%. The concentration of Lome was determined by constructing the calibration curve by diluting 500 ppm of antibiotic solution using the external standard method.

The Orbitrap MS system was tuned and calibrated in positive mode with a standard mixture of caffeine (Mr 194.1 Da), MRFA peptide (Mr 423.6 Da), and Ultramark (Mr 1621 Da). Data acquisition was performed using the Exactive 1.1 SP6 and Thermo Xcalibur 3.0.63 softwares (Thermo Fisher Scientific, San Jose, CA, USA). The chemical structures present in graphs and tables were prepared using the ACD/ChemSketch 2022.2.0 software.

### 2.1. Synthesis of Imprinted Hydro Sample (Imp.Hydro)

A water solution of HEMA and MBA (10% wt.) in a 6:1 ratio was prepared, and once solubilized in the monomer mixture, ammonium persulfate (APS) and N, N, N, N′-tetramethylethylenediamine (TEMED) were added to obtain a concentration of 1%wt, in the final reaction mixture. After 30–40 min, the hydrogel was obtained and used as a reference. 

The imprinted hydrogel was synthesized using the ratio of the same ingredients in a saturated solution of Lomefloxacin or Ciprofloxacin (the templates). After 40 min, the hydrogel was obtained, and sonication was stopped. The resulting products were left overnight in the reaction batch. Afterward, hydrogels were washed at least three times with water and freeze-drying to allow their grinding and chemical–physical characterization. Part of the powder was re-immersed in water and continuously shaken, frequently substituting the solution with fresh water, up to the complete release of templating molecules (2 days). The Lome/Cipro release was followed by UV-visible measurements up to the complete disappearance of the corresponding signals.

### 2.2. Functionalization of Magnetite (Fe_3_O_4_) with Silane Derivative

The Stöber method [24] was adapted to silanize the magnetite with the acrylate derivative. In a 200 mL beaker, 1 g of magnetite nanopowders was suspended in a 50 mL mixture of water/ethanol 70/30 *v*/*v*. This suspension was sonicated using an Ultrasonic Probe Sonicator. The pH was led to 9 using an ammonia solution, and subsequently, 40 mmol of 3-(trimethoxysilyl)propyl methacrylate was added to the reaction mixture, maintaining the sonication for 30 min. The precipitate was washed with water at least three times and dried under a vacuum. 

### 2.3. Synthesis of Magnetic Hydrogel (HydroMAG) 

A total of 60 mg of functionalized magnetite was mixed with a water solution of HEMA and MBA (10% wt.) in a 6:1 ratio under sonication. APS and TEMED were added to obtain a concentration of 1% wt. in the final reaction mixture. 

After 40 min, the hydrogel was achieved. It was shaken in water overnight to eliminate unreacted monomers and nanoparticles and dried in a vacuum oven at 60 °C. 

### 2.4. Synthesis of Molecularly Imprinted Magnetic Hydrogels (Imp.HydroMAG)

A total of 100 mg of functionalized magnetite was mixed with a saturated aqueous solution of Lome or Cipro. HEMA and MBA (10% wt.) were added in a 6:1 ratio under sonication. Finally, the addition of APS and TEMED started the radical reaction, leading to the formation of Imp.HydroMAG. Both materials were washed frequently with a water solution to remove the loaded drug for at least three days. The release of templating molecules was monitored up to the disappearance of the Lome/Cipro UV-Vis signal, thus obtaining the desired imprinted samples. Finally, materials were freeze-dried and gently mashed to proceed with their characterization. Toxicity tests, TGA, FTIR, SEM, and TEM analysis were used to characterize the as-prepared dried sample.

## 3. Characterization

### 3.1. Adsorption Tests 

At least 5 mg of adsorbent materials (Imp.Hydro and Imp.HydroMAG) were soaked in 2 mL of solution containing the selected antibiotics at a concentration of 1.5 × 10^−5^ M for up to 24 h. UV-Vis measurements evaluated the adsorption capacity as a function of immersion time. Specifically, the variation in molar absorbance values at 281 nm for Lomefloxacin and 278 nm for Ciprofloxacin was registered. The adsorption profile for each sample was plotted by considering the ratio between the initial concentration (Co) and those measured in solution after adsorption (Ce), calculated by the Lambert and Beer law.

### 3.2. Photodegradation Tests

**Figure 1:**
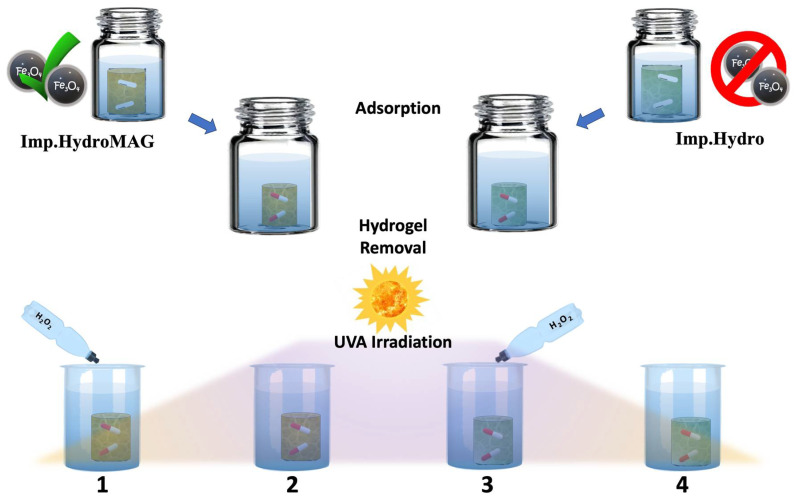
Comparative protocol for verifying the photo degradative capacity of Imp.HydroMAG.

Photo-Fenton process was tested on Lome solution (1.5 × 10^−5^ M) by adding 3 mg of magnetite nanoparticles and 2 mL H_2_O_2_ at 10% *v*/*v* for up to 6 h of UVA exposure with an irradiance of 0.68 W/m^2^/nm (Sample 4, Scheme S1). Similarly, after Lome adsorption in dark conditions, Imp.HydroMAG and Imp.Hydro were recovered, washed with distilled water, and immersed in 2 mL of H_2_O_2_ solution (Sample 1 and 3, Figure 1). After the first oxidation run, the hydrogels were picked up from the solutions and put in fresh water under sonication, repeating the washing procedure at least 10 times. Finally, materials were gently pressed and reused for four consecutive adsorption and photo Fenton cycles. ESI-MS analysis was performed on collected solutions (after appropriate dilution) to determine the occurrence of the photocatalytic degradation process. 

### 3.3. Toxicity Tests

Sample preparation for toxicity testing was described in the experimental section.

Before the toxicity test, the magnetite and freeze-dried hydrogel dry powders were probe-sonicated (4 min at 40 W, 30 °C; Branson Digital Sonifier) in the respective toxicity test medium to obtain aqueous homogenous suspensions. The test medium for the *V. fischeri* assay is 2% NaCl and for the *Daphnia* and algal tests–mineral salts containing media as described in the respective OECD guidelines [25,26]. 

Briefly, in the *D. magna* acute immobilization test (OECD 202, 2004) [25], the actively swimming <24 h old neonates, hatched from the ephippia, were fed with algae *Rapidocelis subcapitata* for two h and then exposed to different nominal concentrations (0.01, 0.1, 1.0, 10, and 100 mg/L) of test materials at 20o C in the dark for 48 h. After 48 h, the number of immobilized (dead) neonates was counted. All samples were analyzed in four replicates in three independent test runs.

The 72 h algal growth inhibition test was performed according to the OECD guideline 201 (OECD, 2011) [26], standard test medium at 24 °C for 72 h, and the initial cell density of *R. subcapitata* was 10^4^ cells/mL. The algae were exposed to the particle suspensions in glass scintillation vials, each containing 5 mL of sample, and vials were shaken on a transparent table illuminated from below at 8000 lux as described in Aruoja et al. [27]. The algae were exposed to different nominal concentrations (0.01, 0.1, 1.0, 10, and 100 mg/L) of test materials in three replicates in two independent test runs. Growth inhibition of algae was calculated from algal biomass that was measured by quantifying the fluorescence of chlorophyll in algal ethanol extracts as described in Joonas et al. [28].

Bacteria *Vibrio fischeri* 30 min acute bioluminescence inhibition assay followed the protocol described in ISO 21338:2010 [29]. *V. fischeri*, also called *Aliivibrio fischeri*, are naturally luminescent marine bacteria that, upon contact with toxic chemicals, decrease their bioluminescence proportionally to the toxicity of this chemical. In contrast, the response is very rapid, within seconds to minutes. The assay was conducted in microplates at 20 °C using Luminometer Orion II (Berthold Detection Systems, Pforzheim, Germany), controlled by Simplicity Version 4.2 Software. Samples were not mixed during the recording of the bacterial luminescence [30]. Different nominal concentrations (0.01, 0.1, 1.0, 10, and 100 mg/L) of test materials in two replicates in two independent test runs were analyzed. 

### 3.4. Calculation of Toxicity Values

EC_50_ or LC_50_ value (mg/L) is the concentration of a material/chemical that either reduces bacterial bioluminescence by 50% (*V. fischeri* assay) or inhibits algal growth by 50% (algal toxicity assay) or immobilizes 50% of daphnids (*D. magna* test). The L(E)C_50_ values were calculated from the respective dose–response curves using REGTOX macro Excel software version 2020 [31] and a log-normal model.

## 4. Results and Discussion

To obtain magnetic hydrogels towards selective antibiotic adsorption, Lome, and Cipro were selected as templating systems.

**Scheme 1:**
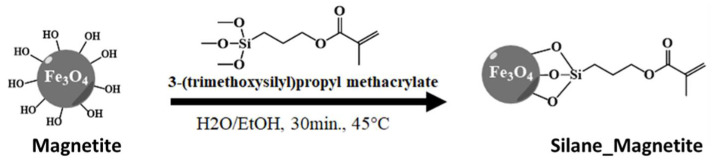
Covalent functionalization of magnetic nanoparticles.

The first step in designing such materials implies the reaction of magnetite nanoparticles with a polymerizable methacrylic silyl derivative (Scheme 1). In this way, the formation of the HEMA/MBA network covalently attached to the magnetite nanoparticles can be achieved.

The proof of the Silane_Magnetite reaction was confirmed by the EDX and FTIR analysis (Figures S1 and S2).

EDX analyses performed on at least three portions of the target sample revealed the appearance of carbon and silicon peaks (Figure S1b) that suggested the success of functionalization.

To further support the formation of a covalent bond between the Si methoxy and the surface exposed OH group of Fe_3_O_4_ nanoparticles, FTIR measurements of the Silane_Magnetite and the pristine magnetite were performed (Figure S2). Interestingly, the signal at 603 cm^−1^ corresponding to the symmetrical stretching of the Fe-O bond of the pristine magnetite (black line), was split into multiple small signals (red line) ascribed to the Si-O-Fe bond, thus confirming the covalent functionalization. In addition, other signals, in the range of 550–500 cm^−1^, might belong to the silane moiety further proving the formation of the desired product. 

To provide the selective sequestering ability toward the antibiotics, Lome or Cipro, respectively, were included in the synthesis of the polymeric network by using saturated solutions of both drugs. Then, they were removed by washing with water at pH ≈ 5 obtaining Imp.HydroMAG (see experimental part). The synthesis of the non-imprinted counterparts (HydroMAG) was carried out to prove the success of the molecular imprinting strategy.

**Figure 2:**
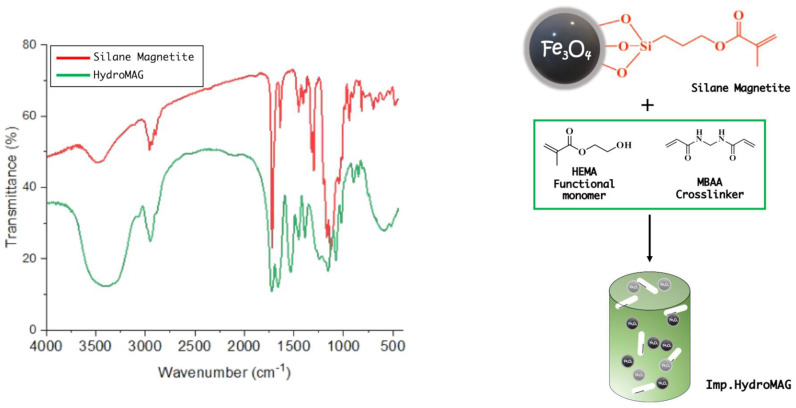
FTIR spectra of silane–functionalized magnetite (red line) and imprinted magnetic hydrogel (green line).

FTIR spectrum of Imp.HydroMAG is reported in Figure 2. It is possible to observe the disappearance of the peak related to monomers’ double bonds (in the range of 1000–800 cm^−1^), which suggests the absence of unreacted monomers [19]. SEM and TGA measurements were also performed (Figures S3 and S4), revealing the typical morphology of hydrogel structures constituted by a large distribution of micropores whose organic part consisted of ~72%. 

### 4.1. Adsorption Tests 

The selective efficiency of imprinted hydrogel in removing Lome or Cipro was evaluated by measuring the absorbance of their solutions (1.5 × 10^−5^ M) before and after MIP immersion for up to 24 h. Additionally, the selectivity of Imp.HydroMAG toward Lome or Cipro was verified by measuring the adsorption of other pollutants [32]; specifically, methylene blue (MB), methyl orange (MO), and 2,4D.

**Figure 3:**
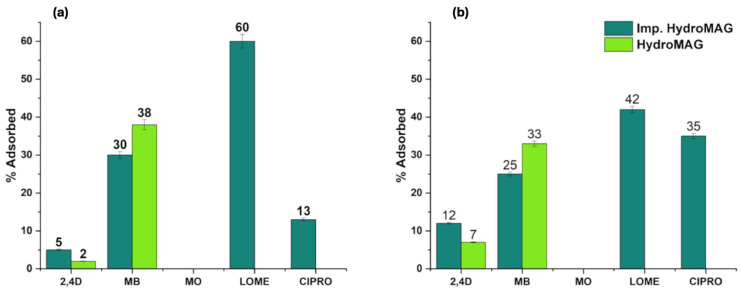
(**a**,**b**) Adsorption performances of HydroMAG and Imp.HydroMAG imprinted with (**a**) Lomefloxacin and (**b**) Ciprofloxacin towards targets (Lome and Cipro, respectively) and different non-target contaminants (2,4-D, MB, and MO).

Figure 3 shows the performed absorbance tests for the Lome-imprinted materials (Figure 3a) and the Cipro-imprinted ones (Figure 3b). The Lome-imprinted material had a clear-cut selectivity towards the target analyte, thus confirming the success of the imprinting process: 60% of the Lome was adsorbed by Imp.HydroMAG. Importantly, the HydroMAG reference sample was inefficient in sequestering Lome (Figure 3a). Adsorbing capacities tested towards 2,4 D, MB, MO, and Cipro showed a clear selectivity of the synthetized materials since the registered depletion of values. On the other hand, the use of Cipro as a templating molecule yielded a selectivity of 35% in Cipro and 42% in Lome (Figure 3b). It is reasonable to suppose that the presence of cyclopropyl and/or the absence of fluoride, if compared to the Lome structure, determine a decrease in the imprinting affinity by the HEMA/MBA network.

Thus, the most efficient MI sample regarding adsorption selectivity was Lome Imp.HydroMAG (Figure 3a), and therefore it was further investigated by TEM and subjected to the PF oxidative process.

### 4.2. TEM Analysis

**Figure 4:**
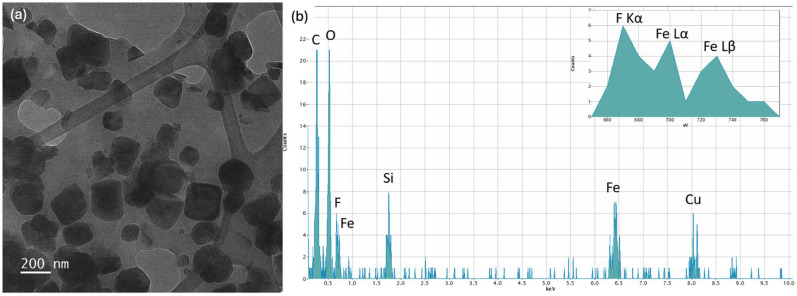
(**a**) BF TEM image of Fe_3_O_4_ nanoparticles after the polymerization which led to the inclusion of the antibiotic; (**b**) relative EDS spectrum with inset highlighting the presence of F Kα peak at 697 eV due to the antibiotic.

Figure 4 reports the TEM analyses of Imp.HydroMAG with Lomefloxacin. Figure 4a shows a BF image of Fe_3_O_4_ nanoparticles after the MI polymerization, which led to the inclusion of the antibiotic in the polymeric coating surrounding the particles, which have a lateral size of tens of nanometers. Figure 4b shows the relative EDS spectrum, which shows carbon, oxygen, fluorine, silicon, and iron peaks coming from the ensemble of polymer, nanoparticles, and antibiotic. Copper peak is due to the TEM grid. Inset of Figure 4b reports in detail the EDS spectrum in the energy range between 650 and 770 eV. Here, along the two Lα and Lβ iron peaks, the presence of the Kα fluorine peak at 697 eV is characteristic of the antibiotic. 

**Figure 5:**
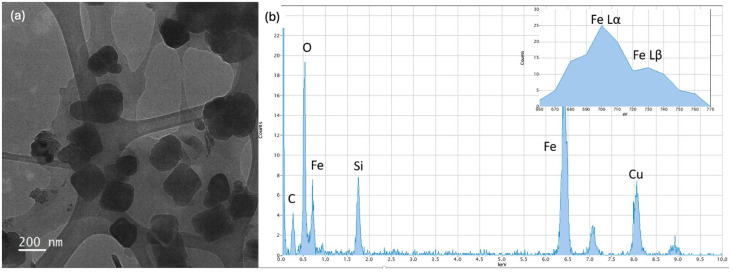
(**a**) BF TEM image of the imprinted Fe_3_O_4_ nanoparticles obtained after the antibiotic removal; (**b**) relative EDS spectrum with inset highlighting the absence of F Kα peak at 697 eV, confirming the physical removal of the antibiotic.

Figure 5a shows the BF image of the imprinted Fe_3_O_4_ nanoparticles obtained after the antibiotic removal. Figure 5b reports the relative EDS spectrum, which shows no more the F peak: differently than the previous figure, the inset of Figure 5b shows only the Lα and Lβ iron peaks characteristic of the nanoparticles, highlighting the absence of the Kα fluorine signal, which is significant for the successful removal of the antibiotic molecules from the polymer coating of the nanoparticles.

### 4.3. Photocatalytic Degradation Tests of Lome

It was proven that Lome can be successfully degraded by photocatalytic systems [33,34] including PF reaction [35]. Preliminary tests on Lome degradation were carried out by employing our UVA 340 lamp irradiation setup. To discern between the different contributions (H_2_O_2_, magnetite, and UVA irradiation) to the eventual degradation of Lome molecules, different sets of references were tested and quantified by ESI-MS, as described in Scheme S1.

The Lome molecules subjected to light irradiation (Sample 1, Scheme S1) were slightly degraded by forming characteristic peaks at *m*/*z* 368.11, 386.12, 308.12, and 332.14 assigned to photooxidation products reported in Table S1 (Figure S6a). By adding magnetite, the degradation was most pronounced. In such a case, as calculated by applying the calibration curve (Figure S5), the decrement of Lome in the solution was about 70%. However, Lome was converted into aromatic degradation products (*m*/*z* 294.12; 308,14; 332.14; and 368.11) whose abundances had comparable orders of magnitude (Sample 3, Scheme S1, Figure S6b). The mass spectrum registered after the PF process (magnetite, H_2_O_2_, and UVA light) confirmed the quasi-total degradation of the drug (Sample 4, Table S1, Figure S6c). As shown in Figure S6c, a complete disappearance of its characteristic peak at *m*/*z* 352.12 was observed. In the same spectrum, it was evident that the formation of degradation products at a lower mass range (Table S1) had a relative abundance four orders of magnitude lesser than the initial concentration of Lome.

Photocatalytic degradation protocol was adapted to MI materials. MI hydrogels with and without magnetite were immersed overnight with the Lome solutions (1 × 10^−5^ M) to reach the adsorption/desorption equilibrium (see experimental part). After that, samples were washed, immersed in distilled water or H_2_O_2_ solution, and subjected to UVA irradiation for up to 6 h, as depicted in Figure 1.

**Figure 6:**
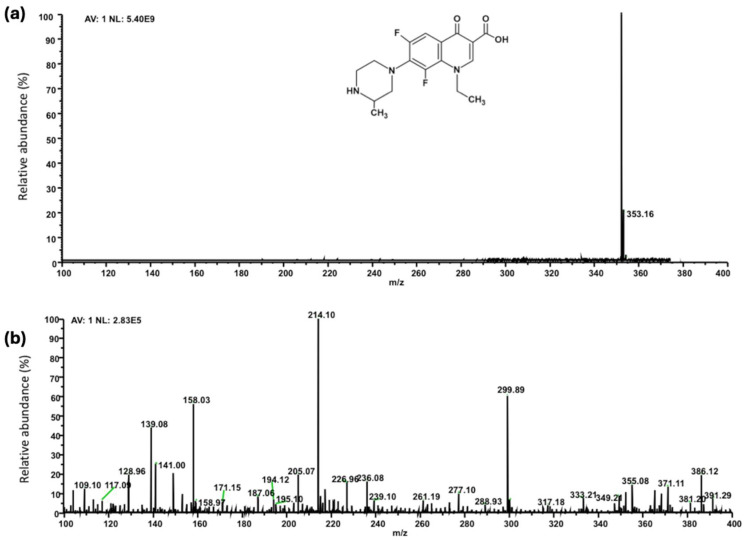
(**a**,**b**) ESI MS of (**a**) Lomefloxacin solution (Sample 5, Scheme S2), and (**b**) after UVA exposure in presence of Imp.HydroMAG.

Collected solutions deriving from Samples 1–4 (Figure 1) were characterized by ESI-MS analysis. Figure 6a shows the mass spectrum of the Lome solution (Sample 5, Scheme S1). As shown in Figure 6b, the photo irradiation of Imp.HydroMAG in the presence of hydrogen peroxide (Sample 1) caused the disappearance of the Lome signal, which agrees with the results reported above. Additionally in this case, the mass spectrum reveals the presence of degradation products with a concentration of four orders of magnitude lesser than the C_0_, corroborating previously obtained data, thus confirming the efficacy of Imp.HydroMAG in mineralizing the target molecule.

To test the reusability, Imp.HydroMAG was subjected to four runs of adsorption and photocatalytic runs (Figure S7) showing that the material can be reused for up to three cycles without a lack of performance. However, as shown from the ESI spectrum (Figure S8) of the solution collected at the fourth run, the signal of Lome appeared, evidencing the reduction efficiency of the PF process in degrading the Lome molecules.

### 4.4. Toxicity Evaluation of the Studied Materials

Before allowing novel materials to the market, data on its environmental safety are crucial because all materials will sooner or later end up in the environment, usually via various waste streams of the materials’ life cycle. To address that aspect, the ecotoxicity of the synthesized materials was analysed using a battery of three toxicity assays of environmentally relevant test species of different food web levels: bacteria, crustaceans, and algae. The assays were (i) 30 min *Vibrio fischeri* bioluminescent inhibition assay (ISO 21338:2010) [29], (ii) 48 h *Daphnia magna* immobilization test (OECD 202, 2004) [25], and (iii) 72 h *Raphidocelis subcapitata* growth inhibition test (OECD 201, 2011) [26]. 

It is important to note that tests with algae (usually *Rapidocelis subcapitata* as a proxy organism for primary producers) and aquatic crustaceans (usually *Daphnia magna* as a proxy for primary consumers) are required in REACH regulation [5] for environmental hazard evaluation of chemicals produced from 1 to 10 tons annually. In addition, naturally luminescent marine bacteria *Vibrio fischeri* are also environmentally relevant test organisms and can be considered as a proxy for degraders/decomposers, which is a very important organism group in ecosystems. 

In terms of ecosafety evaluation, it is generally considered that when the chemical/material at a concentration of 100 mg/L is not inhibitory to any test organism in the above described test battery (i.e., L(E)C_50_ value > 100 mg/L), it can be considered not harmful to the aquatic ecosystems. In case of L(E)C_50_ values in the range of 0.01 mg/L up to >100 mg/L, the following evaluation grid applied by Sanderson et al. [36] and Blaise et al. [37], was suggested and was also used in the current paper. This classification is based on the L(E)C_50_ value of the most sensitive organism used: ≤0.1 mg/L = extremely toxic to aquatic organisms; >0.1–1 mg/L = very toxic to aquatic organisms; >1–10 mg/L = toxic to aquatic organisms; >10–100 mg/L = harmful to aquatic organisms; and >100 mg/L = non-toxic/non-harmful to aquatic organisms. 

Magnetite and altogether five different synthesized imprinted and not imprinted samples were analysed for the ecotoxicity (Table 1): hema hydrogel (Hema Hydro), hema hydrogel imprinted with Lomefloxacin (Imp.Hydro with Lome), imprinted hema hydrogel (Imp.Hydro), HydroMag, Imp.HydroMAG with Lome.

In addition, ZnSO_4_ was used as a positive control, and as expected, Zn^2+^ proved toxic to bacteria *V. fischeri* (EC_50_ 5.9 mg Zn/L), to crustaceans *D. magna* (LC_50_ 1.4 mg Zn/L), and was extremely toxic to algae *R. subcapitata* (EC_50_ 0.08 mg Zn/L). These data are coherent with the toxicity values for Zn^2+^ obtained by other researchers [38] and prove the validity of the toxicity test data.

Toxicity data obtained are listed in Table 1 and show that magnetite (Sample 1 in Table 1) was not toxic to bacteria *V. fischeri* (30 min EC_50_ > 100 mg/L) and crustaceans *D. magna* (48 h LC_50_ > 100 mg/L), but remarkably inhibited the growth of algae (72 h EC_50_ = 2.2 mg/L). That would rank magnetite as ‘toxic’. However, synthesized samples with no magnetite in their composition (Samples 2, 3, and 4 in Table 1) showed no toxic effects on algae, crustaceans, and bacteria (EC_50_ > 100 mg/L), and could be thus considered as not toxic to the aquatic ecosystem. Expectedly, the magnetite-containing materials HydroMAG and Imp.HydroMAG with Lomefloxacin (Samples 5 and 6 in Table 1), although not toxic to bacteria (EC_50_ > 100 mg/L) and daphnids (EC_50_ > 100 mg/L), proved harmful (EC_50_ 30.7 mg/L) to algae. As magnetite was the toxic component of this composite to algae (Sample 1; EC_50_ 2.2 mg/L, i.e., 1–10 mg/L), the addition of organic components “diluted” or “screened” the toxic effect of magnetite to algae. Indeed, the HydroMAG was already less toxic than magnetite (EC_50_ 13.8 mg/L versus EC_50_ 2.2 mg/L) and the final product Imp.HydroMAG with Lomefloxacin is in turn less toxic than the final composite Imp.HydroMAG with Lomefloxacin (EC_50_ 30.7 mg/L) (Table 1). 

Thus, one may suggest that the composites with magnetite must be handled with care, and release to aquatic water bodies must be minimized.

## 5. Conclusions

The development of materials capable of reducing water contamination from emerging pollutants is a challenging task. This complexity stems from the need to selectively remove trace amounts of molecules from the contaminated site while ensuring that photocatalytic oxidation processes do not introduce secondary pollutants, such as nanoparticles, that undermine the desired effect. Additionally, industrial applications require low-cost and eco-sustainable materials and processes. In this work, we demonstrated that the formulation of molecular imprinted polymer (MIP) materials containing magnetite covalently linked to the matrix can selectively adsorb Lomefloxacin and degrade it through the photo-Fenton reaction. Notably, this approach prevents the release of nanoparticles into the environment during the oxidation process. Since Imp.HydroMAG can inhibit algal growth to some extent, its release into aquatic environments should be minimized. Furthermore, the hydrogel formulation allows for easy regeneration of the freestanding catalyst, which can be reused for up to four cycles. More importantly, this design can be reasonably extended to selectively sequester other contaminants by modifying the MIP synthesis, thereby opening new possibilities in the formulation of materials for water remediation.

## Figures and Tables

**Table 1:** Toxicity, L(E)C values (mg/L) of studied materials imprinted with Lomefloxacin (Lome) molecules.

	Test *Organism*	Bacteria *Vibrio fischeri*	Crustaceans *Daphnia magna*	Algae *Raphidocelis subcapitata*
	Toxicity endpoint, contact time:	Inhibition of luminescence, 30 min	Immobilisation, 48 h	Inhibition of growth, 72 h
1	Magnetite (Fe_3_O_4_)	Not toxic (EC_50_ > 100 mg/L)	Not toxic (LC_50_ > 100 mg/L)	Toxic (EC_50_ 2.2 mg/L)
2	HEMA Hydro	Not toxic (EC_50_ > 100 mg/L)	Not toxic (LC_50_ > 100 mg/L)	Not toxic (EC_50_ > 100 mg/L)
3	Imp.Hydro + Lome	Not toxic (EC_50_ > 100 mg/L)	Not toxic (LC_50_ > 100 mg/L)	Not toxic (EC_50_ > 100 mg/L)
4	Imp.Hydro	Not toxic (EC_50_ > 100 mg/L)	Not toxic (LC_50_ > 100 mg/L)	Not toxic (EC_50_ > 100 mg/L)
5	HydroMAG	Not toxic (EC_50_ > 100 mg/L) Inhibition at 100 mg/L less than 5%	Not toxic (EC_50_ > 100 mg/L) Immobilisation at 100 mg/L–0	Harmful (EC_50_ 13.8 mg/L)
6	Imp.HydroMAG + Lome	Not toxic (EC_50_ > 100 mg/L) Inhibition at 100 mg/L less than 5%	Not toxic (EC_50_ > 100 mg/L) Immobilisation at 100 mg/L–0	Harmful (EC_50_ 30.7 mg/L)
8	ZnSO_4_x7H_2_O, Alfa Aesar (positive control)	Toxic EC_50_ = 5.9 mg Zn/L	Toxic LC_50_ = 1.4 mg Zn/L^3^	Extremely toxic EC_50_ = 0.08 mg Zn/L

≤0.1 mg/L = extremely toxic to aquatic organisms; >0.1–1 mg/L = very toxic to aquatic organisms; >1–10 mg/L = toxic to aquatic organisms; >10–100 mg/L = harmful to aquatic organisms; and >100 mg/L = non-toxic to aquatic organisms. The evaluation grid applied by Sanderson et al. [36].

## Data Availability

Data are contained within the article and Supplementary Materials.

## Supplementary Materials

The following supporting information can be downloaded at: https://www.mdpi.com/article/10.3390/polym16152096/s1, Figure S1: (a) SEM image and (b) EDX analysis of the functionalized Fe_3_O_4_ nanoparticles; Figure S2: FT-IR spectra of Silane_Magnetite (red profile) and Fe_3_O_4_ (black line). Figure S3: (a) TGA and DTG of Imp.HydroMAG. Analysis was carried out under nitrogen flux. Thermogravimetric analysis was performed to obtain indications of the thermal behavior of the sample and to gather information on the residue and chemical composition. The thermogram of Imp.HydroMAG shows a single degradation step with a maximum degradation rate (T_max_) at 412.37 °C, as evident from the DTG (derivative thermogravimetric) profile. The residue is stable up to 700 °C and is consistent with the inorganic content of the hybrid material (Fe_3_O_4_ nanoparticles); Figure S4: (a) SEM Image; (b) Porosimetry graphical analysis; (c) circle equivalent diameter plot, and d) Pore Properties Table of the HydroMAG. Figure S5: (a) Lomefloxacin calibration curve obtained by ESI MS analysis. Figure S6: (a) ESI-MS spectrum of (a) sample 1; (b) sample 3; and (c) Sample 4 (Scheme S1). Figure S7: Recyclability of the material after four adsorption cycles. Figure S8: (a) ESI-MS spectrum of (a) Sample 2; (b) Sample 3 as described in Figure 2, and (c) solution collected after the 4th regeneration run (Figure S7). Figure S9: Size distribution histogram obtained using the “particle analysis” function of Digital Micrograph software on 580 nanoparticles. Scheme S1: Graphic resume of analyzed solutions. Specifically, sample 1 is the Lome solution subjected to UVA irradiation; sample 2 is the Lomefloxacin solution with H_2_O_2_ under UVA irradiation; sample 3 is Lome solution UVA-irradiated in the presence of Fe_3_O_4_; sample 4 is the Lome solution under UVA irradiation in the presence of H_2_O_2_ and Fe_3_O_4_. Samples 5–8 correspond to 1–4 placed in dark conditions. Scheme S2: Temporal representation of the cryogel synthesis experiment, production of the hybrid, adsorption of Lomefloxacin, and exposure to the UV lamp for photodegradation of the drug. Table S1: Proposed peak assignments belonging to degradation intermediates of Lome during the whole reaction.; Video S1: Magnetic Hydrogel.
