# Supplementary material for: Design of High-Performance Molecular Imprinted Magnetic Nanoparticles-Loaded Hydrogels for Adsorption and Photodegradation of Antibiotics from Wastewater
Source: Polymers (Basel). 2024 Jul 23;16(15):2096. doi: 10.3390/polym16152096 (PMC11314038; doi:10.3390/polym16152096)
Supplement: Supplementary file 1 [file polymers-16-02096-s001.zip › polymers-3104803-supplementary.pdf]

Supplementary Material

Design of High-Performance Molecular Imprinted Magnetic Nanoparticles-Loaded Hydrogels for Adsorption and Photodegradation of Antibiotics from Wastewater

Giusy Curcuruto [1], Andrea A. Scamporrino [1,*], Roberta Puglisi [1,2], Giuseppe Nicotra [3], Gianfranco Sfuncia [2], Giuliana Impellizzeri [4], Sandro Dattilo [1], Anne Kahru [5,*], Mariliis Sihtmäe [5], Villem Aruoja [5], Irina Blinova [5] and Sabrina Carola Carroccio [1]

[1] Institute for Polymers, Composites, and Biomaterials CNR-IPCB, Via Paolo Gaifami 18, 95126 Catania, Italy; giusy.curcuruto@cnr.it (G.C.); roberta.puglisi@unict.it (R.P.); sandro.dattilo@cnr.it (S.D.); sabrinacarola.carroccio@cnr.it (S.C.C.)
[2] Department of Chemical Sciences, University of Catania, Viale Andrea Doria 6, 95125 Catania, Italy; gianfranco.sfuncia@cnr.it
[3] Institute for Microelectronics and Microstructures CNR-IMM, Zona Industriale Strada VIII, 5, 95121 Catania, Italy; giuseppe.nicotra@cnr.it
[4] Institute for Microelectronics and Microstructures CNR-IMM, Via Santa Sofia 64, 95123 Catania, Italy; giuliana.impellizzeri@ct.infn.it
[5] Laboratory of Environmental Toxicology, National Institute of Chemical Physics and Biophysics, Akadeemia tee 23, 12618 Tallinn, Estonia; mariliis.sihtmae@kbfi.ee (M.S.); villem.aruoja@kbfi.ee (V.A.); irina.blinova@kbfi.ee (I.B.)
* Correspondence: andreaantonio.scamporrino@cnr.it (A.A.S.); anne.kahru@kbfi.ee (A.K.)

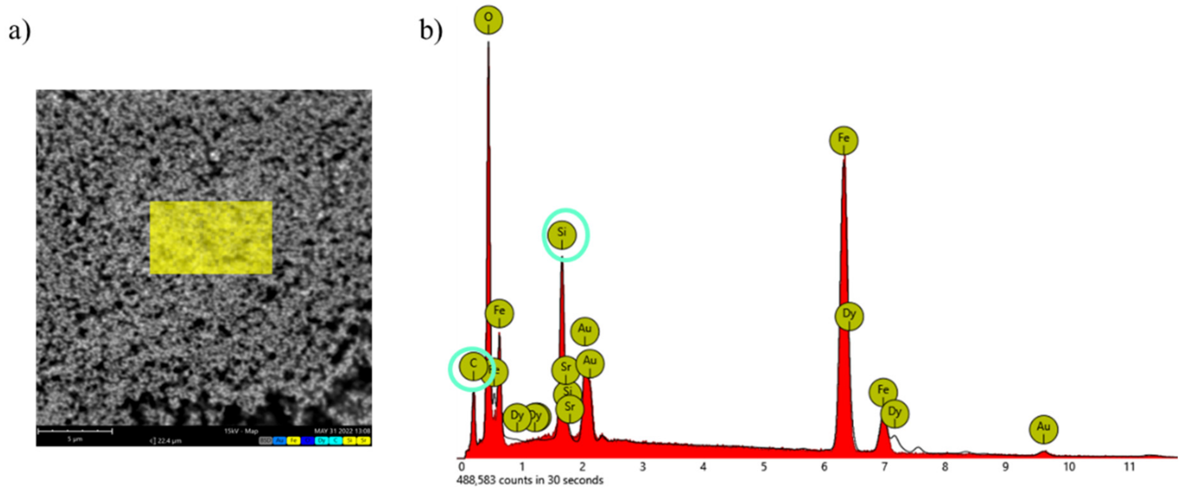

Figure S1. a) SEM image and b) EDX analysis of the functionalized Fe_3O_4 nanoparticles.

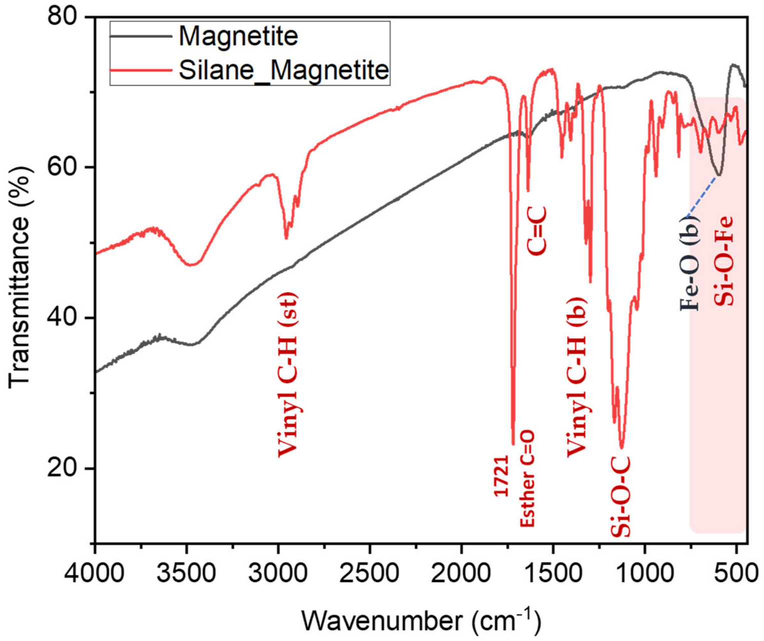

Figure S2. FT-IR spectra of Silane_Magnetite (red profile) and Fe_3O_4 (black line).

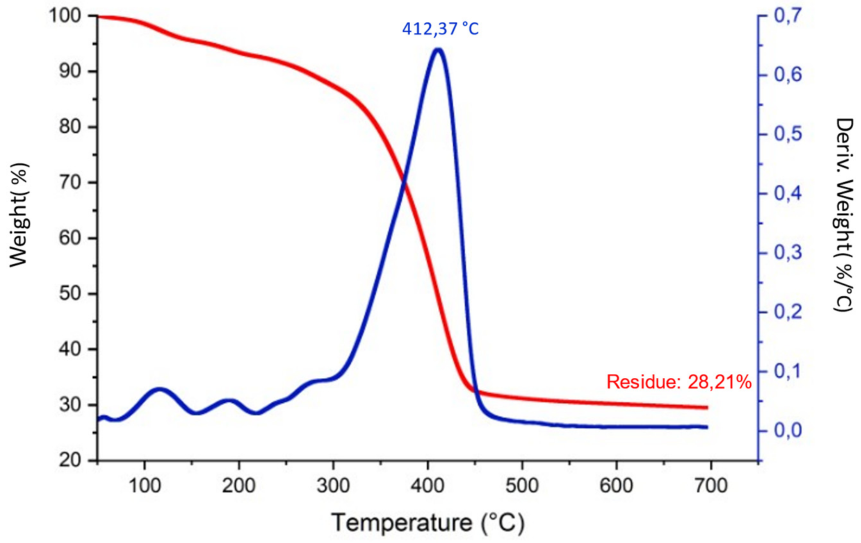

Figure S3. a) TGA and DTG of Imp.HydroMAG. Analysis was carried out under nitrogen flux.
Thermogravimetric analysis was performed to obtain indications of the thermal behavior of the sample and to gather information on the residue and chemical composition. The thermogram of Imp.HydroMAG shows a single degradation step with a maximum degradation rate (T_{max}) at 412.37 °C, as evident from the DTG (derivative thermogravimetric) profile. The residue is stable up to 700 °C and is consistent with the inorganic content of the hybrid material (Fe_3O_4 nanoparticles).

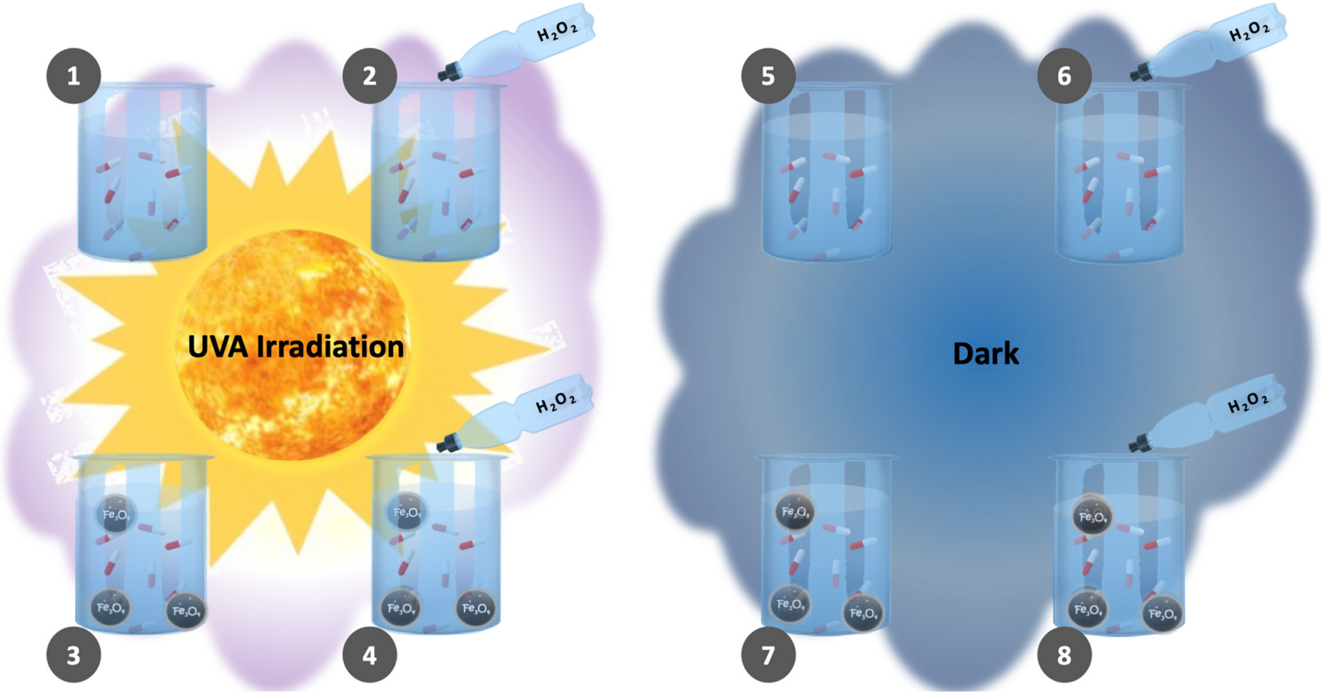

Scheme S1. Graphic resume of analyzed solutions. Specifically, sample 1 is the Lome solution subjected to UVA irradiation; sample 2 is the Lomefloxacin solution with H_2O_2 under UVA irradiation; sample 3 is Lome solution UVA-irradiated in the presence of Fe_3O_4; sample 4 is the Lome solution under UVA irradiation in the presence of H_2O_2 and Fe_3O_4. Samples 5-8 correspond to 1-4 placed in dark conditions.

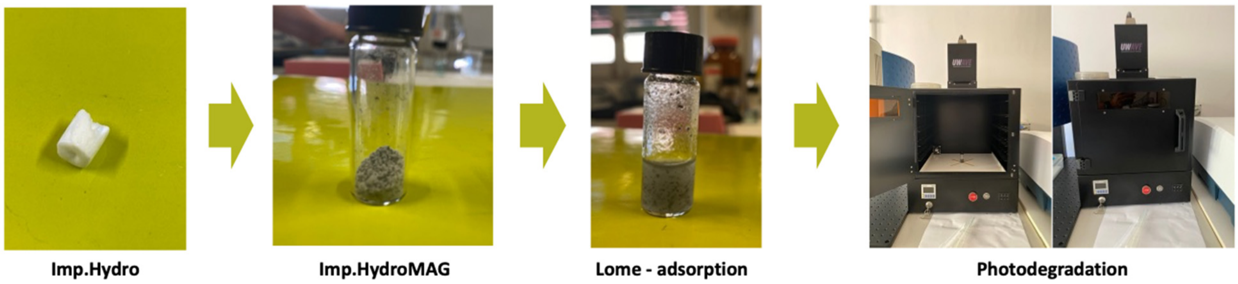

Scheme S2. Temporal representation of the cryogel synthesis experiment, production of the hybrid, adsorption of Lomefloxacin, and exposure to the UV lamp for photodegradation of the drug.

Table S1. Proposed peak assignments belonging to degradation intermediates of Lome during the whole reaction.

Sample	m/z	Formula	Possible Structure
P1	386.12	$C_{16}H_{17}F_2N_3O_6$	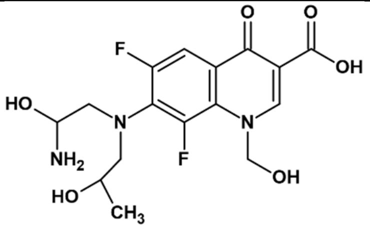
P2	368.11	$C_{17}H_{19}F_2N_3O_4$	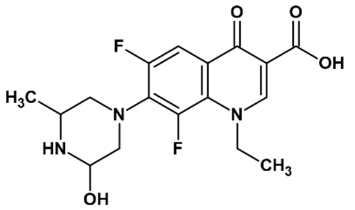
P3	352.14	$C_{17}H_{19}F_2N_3O_3$	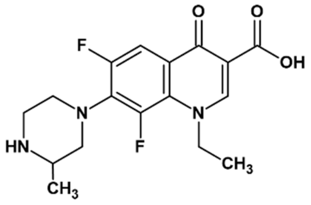
P4	332.14	$C_{17}H_{21}FN_3O_4$	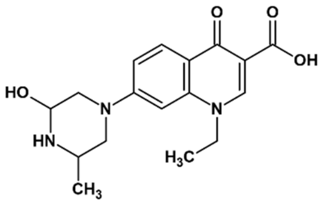
P5	308.14	$C_{15}H_{18}FN_3O_3$	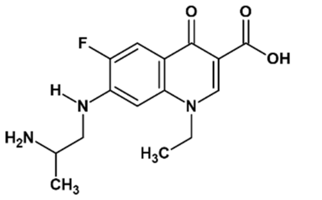
P6	294.12	$C_{14}H_{16}FN_3O_3$	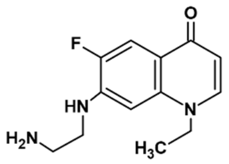
P7	139.08	$C_7H_6O_3$	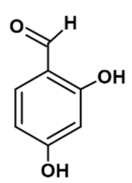

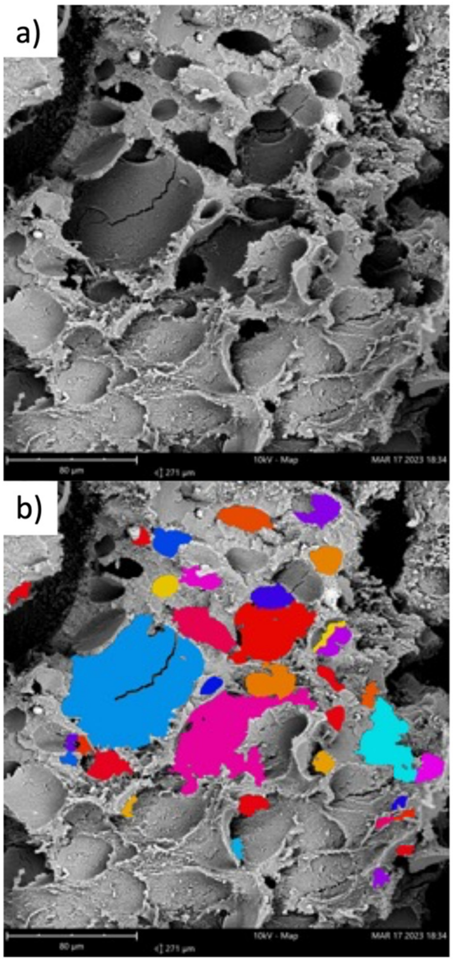

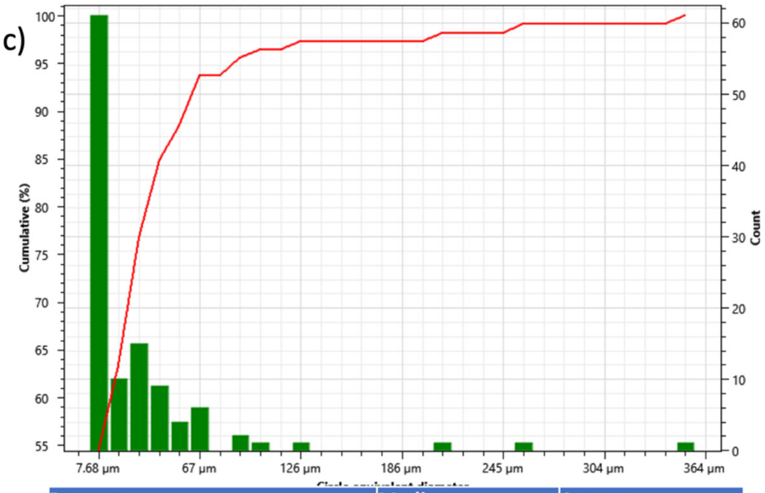

d)

Property	Median	Average
Circle equivalent diameter	18.3 µm	35.3 µm
Major axis	23.9 µm	51.1 µm
Minor axis	14.4 µm	25.4 µm
Circumference	71.9 µm	171 µm
Convex hull	66.4 µm	142 µm
Circumscribed circle diameter	26.9 µm	59.2 µm
Area	262 µm²	2.8E+03 µm²
Volume by area	3.19E+03 µm³	4.01E+05 µm³
Pixel count	1652	4815
Aspect ratio	0.566	0.575
Circularity	0.59	0.592
Convexity	0.939	0.909
Elongation	0.434	0.425
Grayscale	203	206
Inscribed circle diameter	13.4 µm	21.8 µm

Figure S4. a) SEM Image; b) Porosimetry graphical analysis; c) circle equivalent diameter plot, and d) Pore Properties Table of the HydroMAG.

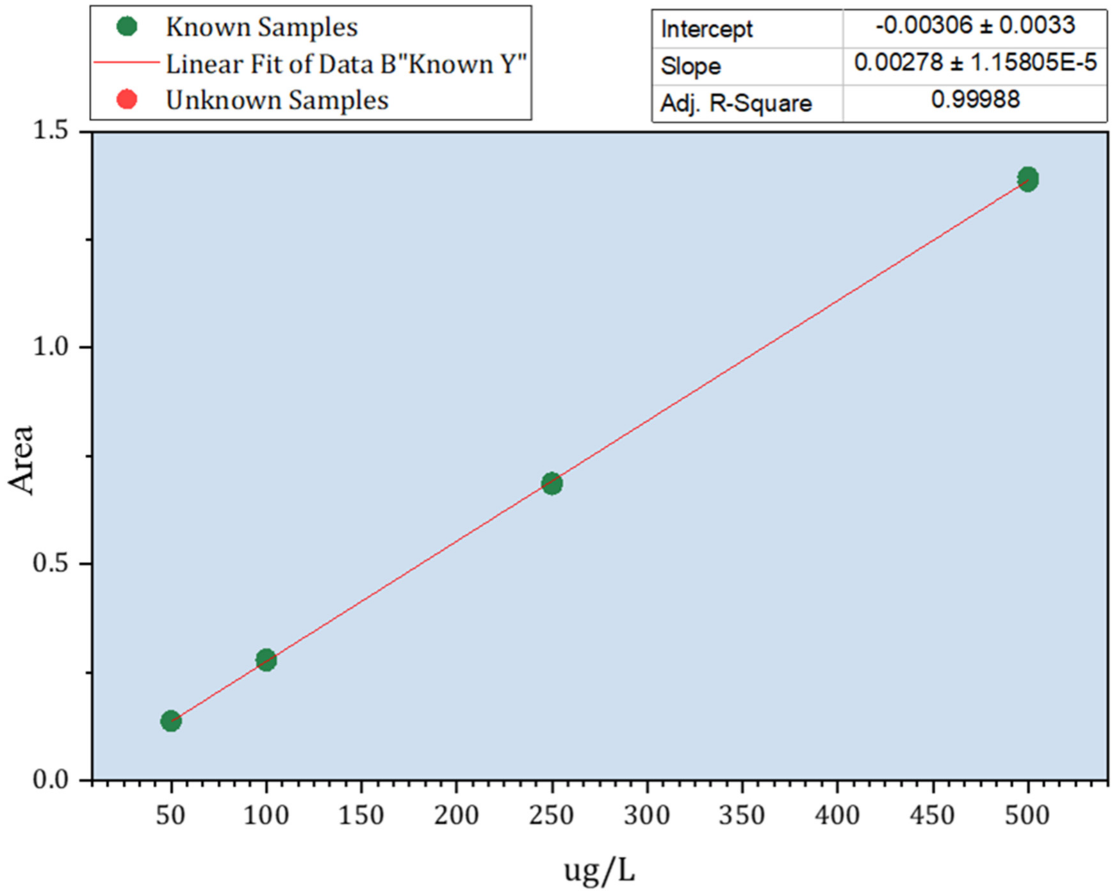

Figure S5. a) Lomefloxacin calibration curve obtained by ESI MS analysis.

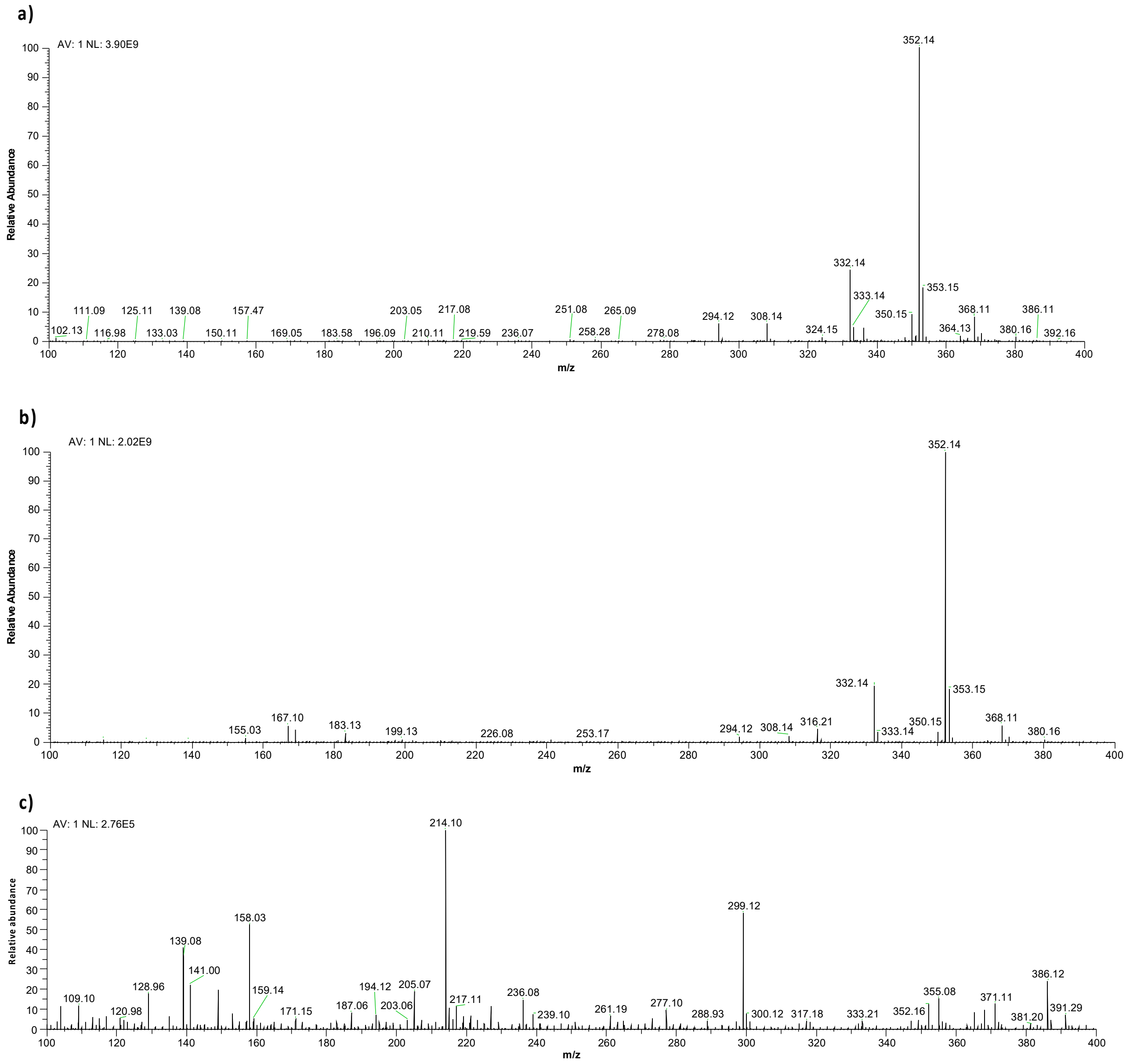

Figure S6. a) ESI-MS spectrum of a) sample 1; b) sample 3; and c) Sample 4 (Scheme S1)

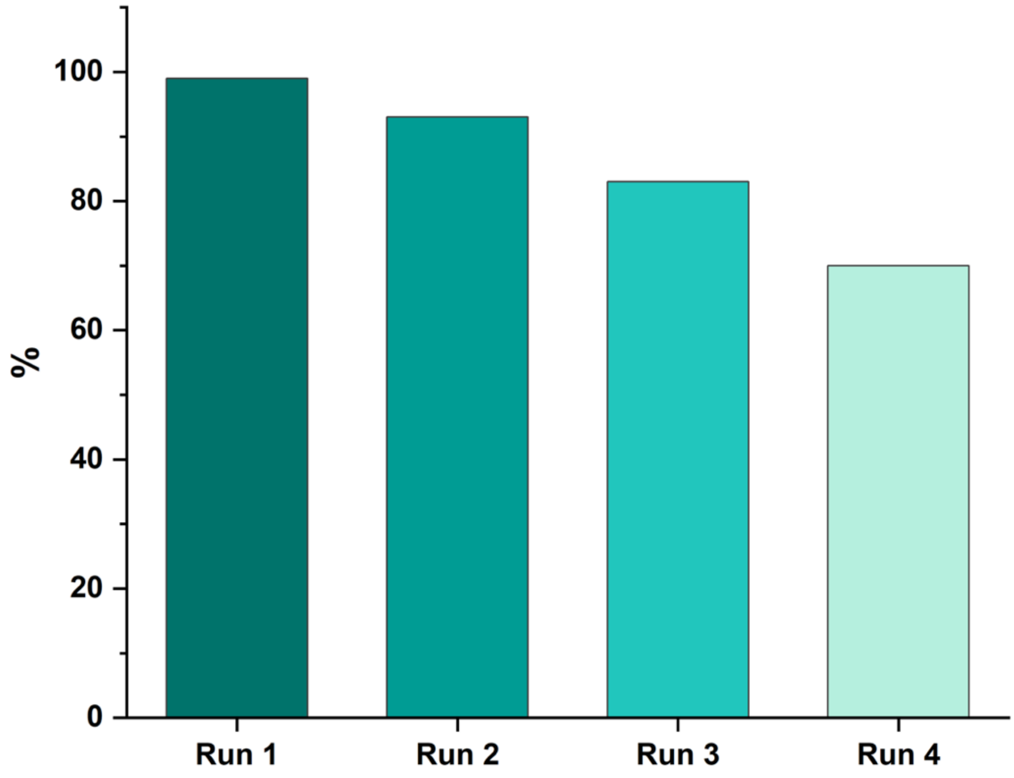

Figure S7. Recyclability of the material after four adsorption cycles.

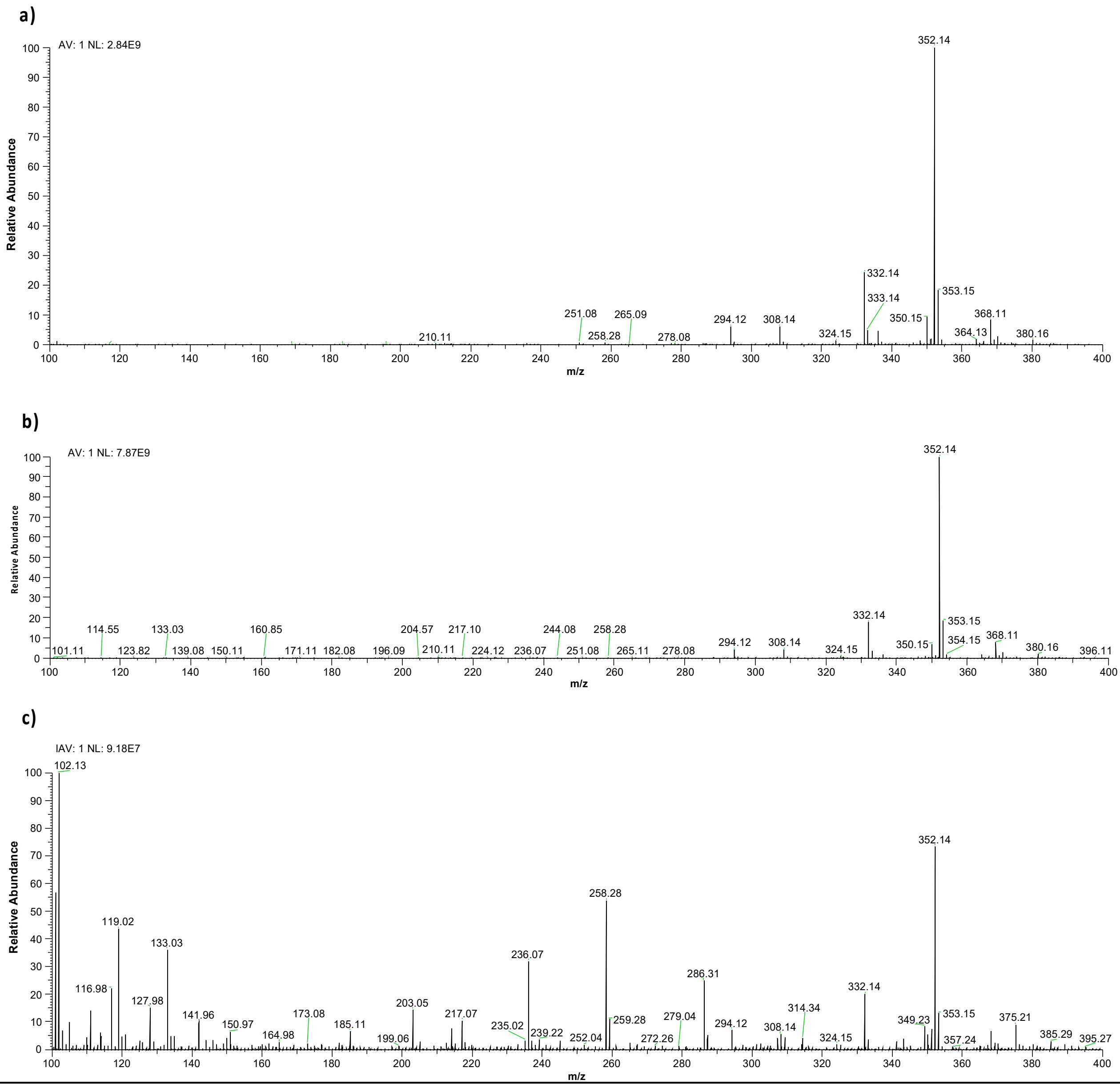

Figure S8. a) ESI-MS spectrum of a) Sample 2; b) Sample 3 as described in Figure 2, and c) solution collected after the 4th regeneration run (Figure S7).

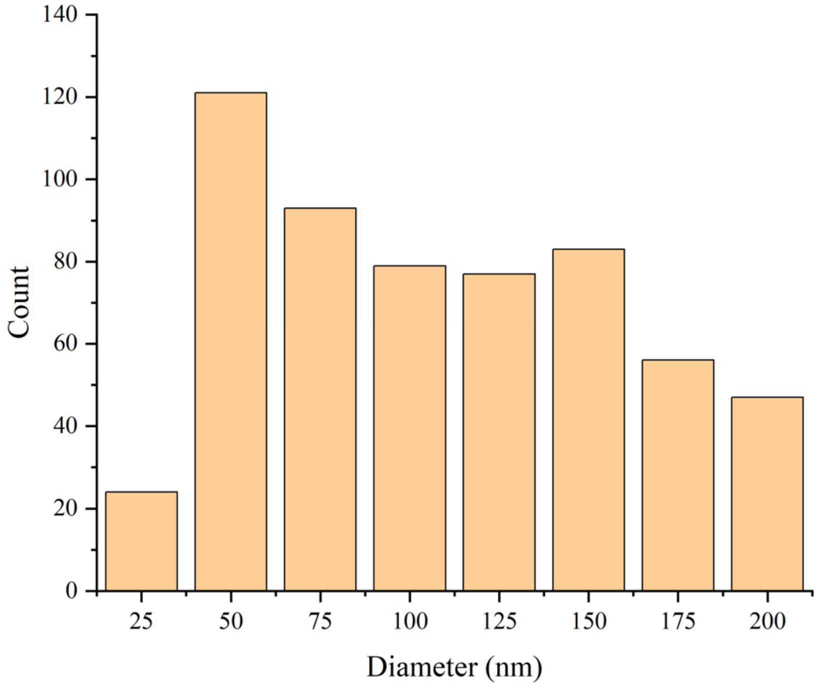

Figure S9. Size distribution histogram obtained using the "particle analysis" function of Digital Micrograph software on 580 nanoparticles.
